# Superior cyclability of high surface area vanadium nitride in salt electrolytes†

**DOI:** 10.1039/d2na00810f

**Published:** 2023-06-09

**Authors:** James Kasten, Cheng-Che Hsiao, Denis Johnson, Abdoulaye Djire

**Affiliations:** a Artie McFerrin Department of Chemical Engineering, Texas A&M University College Station TX 77843 USA; b Department of Materials Science & Engineering, Texas A&M University College Station TX 77843 USA adjire@tamu.edu

## Abstract

High surface area vanadium nitrides (VNs) have been extensively studied as materials for aqueous supercapacitors due to the high initial capacitance in alkaline media at low scan rates. However, low capacitance retention and safety limit their implementation. The use of neutral aqueous salt solutions has the potential to mitigate both of these concerns, but is limited in analysis. Hence, we report on the synthesis and characterization of high surface area VN as a supercapacitor material in a wide variety of aqueous chlorides and sulfates using Mg^2+^, Ca^2+^, Na^+^, K^+^, and Li^+^ ions. We observe the following trend in the salt electrolytes: Mg^2+^ > Li^+^ > K^+^ > Na^+^ > Ca^2+^. Mg^2+^ systems provide the best performance at higher scan rates with areal capacitances of 294 μF cm^−2^ in 1 M MgSO_4_ over a 1.35 V operating window at 2000 mV s^−1^. Furthermore, VN in 1 M MgSO_4_ maintained a 36% capacitance retention from 2 to 2000 mV s^−1^ compared to 7% in 1 M KOH. Capacitance in 1 M MgSO_4_ and 1 M MgCl_2_ increased to 121% and 110% of their original values after 500 cycles and maintained capacitances of 589 and 508 μF cm^−2^ at 50 mV s^−1^ after 1000 cycles, respectively. In contrast, in 1 M KOH the capacitance decreases to 37% of its original value, reaching only 29 F g^−1^ at 50 mV s^−1^ after 1000 cycles. The superior performance of the Mg system is attributed to a reversible surface 2 e^−^ transfer pseudocapacitive mechanism between Mg^2+^ and VN_*x*_O_*y*_. These findings can be used to further the field of aqueous supercapacitors to build safer and more stable energy storage systems that can charge quicker compared to KOH systems.

## Abstract 2

High surface area vanadium nitride based supercapacitors using aqueous Mg salt electrolytes.

## Graphical abstract

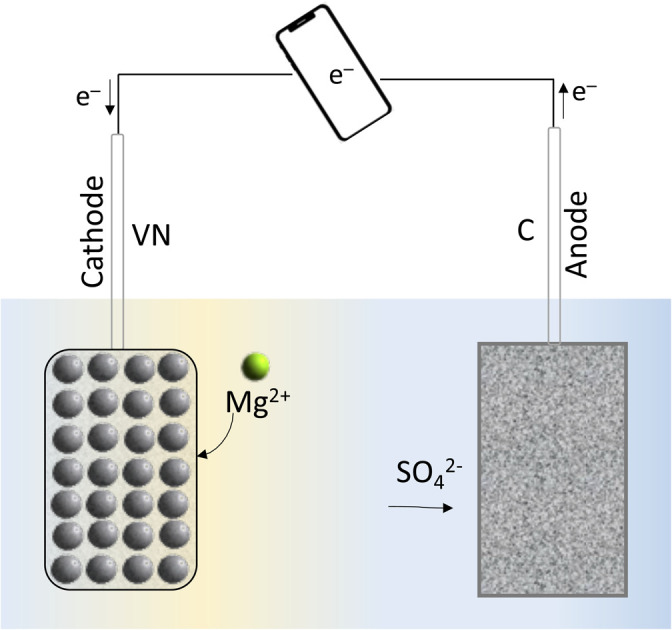

## Introduction

Supercapacitors are electrochemical energy storage devices that have the potential to replace and/or complement batteries for electronics and large-scale energy applications, due to their high power densities (higher than those of batteries) and moderate energy densities.^1–10^ In the search to find cost-effective and efficient materials for supercapacitor use, a wide range of composite materials, including polymers,^11–15^ metal oxides^16–22^ and sulfides,^23–27^ and higher surface area transition-metal carbides^28–33^ and nitrides,^34–37^ are frequently investigated due to their pseudocapacitive charge storage mechanisms involving fast and reversible faradaic redox reactions which contribute to substantially larger capacitances compared to double layer capacitors. Among this material class, vanadium nitride (VN) has been thoroughly investigated and lauded for achieving high capacitances of up to 1340 F g^−1^ in alkaline environments.^38–40^ This high capacitance is attributed to a redox reaction occurring at the oxynitride (VN_*x*_O_*y*_) surface with hydroxy ions as verified by *in situ* analysis of the pseudocapacitive mechanism.^39,41^

However, this impressive capacitance attributed to VN in alkaline systems is exclusively reported at low scan rates of 2 mV s^−1^ due to the significant drop in performance as scan rates increase based on electrolyte concentration. Few literature studies have reported capacitance values beyond 300 mV s^−1^ with capacitance retention dropping to as low as 20% from 2 to 100 mV s^−1^.^38,42–44^ Moreover, at KOH concentrations beyond 1 M, VN exhibits poor voltametric cyclability leading to steep drops in capacitance retention after only a few hundred cycles.^38,45^ Finally, there is a severe dearth of studies reporting on the performance of high surface area VN in non-alkaline, specifically pH-neutral, electrolytes such as aqueous salts.^38,46–48^ Given the highly corrosive nature of high concentration (∼6 M) KOH used in standard electrochemical setups for VN supercapacitors, there is an additional safety concern regarding the highly cathodic potentials being reached during the charging process leading to corrosion and additional maintenance.^42,49,50^ Considering these gaps in KOH systems against a desire to construct supercapacitors with high cyclability and performance at fast charging rates, the practical shortcomings of KOH as a superlative electrolyte for VN supercapacitors become more evident. This serves as an exigence to explore VN in other electrolyte systems which may prove to be more preferential for charging and stability conditions which KOH cannot currently fulfill.

Herein, we report the synthesis and characterization of high surface area VN as a supercapacitor material in a wide variety of aqueous salt electrolytes as well as 1 M and 0.1 M KOH for comparison. The high surface area VN is synthesized *via* a standardized temperature-programmed reduction and nitridation of a V_2_O_5_ precursor in an ammonia (NH_3_) atmosphere and then passivated at room temperature with a surface oxide layer.^51^ X-ray diffraction (XRD) analysis is performed on the bulk to confirm the successful reduction and nitridation of the precursor oxide. N_2_ physisorption is used to analyze and quantify pore size distribution and physical surface area. Raman, X-Ray Photoeletron (XPS), and Fourier-transform infrared (FTIR) spectra are recorded to confirm the successful passivation of the VN by an oxide layer. Electrochemical performance was assessed using cyclic voltammetry (CV) and electrochemical impedance spectroscopy (EIS) of VN in a series of chloride (Cl^−^) and sulfate (SO_4_^2−^) solutions containing Mg^2+^, Ca^2+^, Li^+^, Na^+^, and K^+^ cations in addition to 1 M and 0.1 M KOH for comparison. Aqueous salt systems exhibited similar voltage windows to the KOH systems indicating that they can be used in place of the corrosive alkaline media. Electrochemical performance was determined by evaluating capacitance in each electrolyte against the scan rate and number of cycles. It was found that MgSO_4_ at high scan rates (2000 mV s^−1^) outperformed 1 M KOH indicating higher capacitance for fast charge technologies. It was also found that MgSO_4_ based supercapacitors provide significantly greater stability when compared to the KOH systems. Finally, these data were used to hypothesize a novel pseudocapacitive mechanism of high surface area VN in Mg^2+^ systems.

## Experimental methods

### Material synthesis

Vanadium nitride was synthesized by reducing the precursor V_2_O_5_ (Sigma-Aldrich) by a temperature-programmed reaction (TPR) synthesis at 750 °C in an NH_3_ atmosphere. In brief, the oxide precursor was first ground and sieved to keep the particle size below 37 μm then placed on a quartz boat in a quartz tube reactor. The reactor was ramped up to 750 °C and kept at that temperature for 1 hour. After synthesis, the material was cooled to room temperature followed by flowing a mixture of 1% O_2_/He (Airgas) to intentionally passivate the material, which will prevent the bulk material from further oxidation when exposed to air.

### Physical characterization

The bulk crystalline structure of the material was characterized by X-ray diffraction (XRD) using a Rigaku Miniflex diffractometer. XRD was conducted over a 2*θ* range of 3° to 90° at a scan rate of 2.0° min^−1^. Surface area and pore size distribution were determined by N_2_-physisorption (Quantachrome Autosorb-iQ) with the Brunauer–Emmett–Teller (BET) method and Barrett–Joyner–Halenda (BJH) method, respectively. The material was degassed in vacuum at 350 °C for 8 hours before the measurement. Raman spectroscopy was carried out using a Renishaw inVia Qontor with a 532 nm laser, 1800 lines per mm grating, and a 50× long objective lens. Fourier Transform Infrared spectroscopy (FTIR) was conducted on a Bruker INVENIO R with a diamond ATR module installed.

### Electrode preparation

Electrodes were prepared *via* a slurry method containing 85% VN, 10% carbon black (Super P®, Alfa Aesar) and 5% polyvinyldifluoride (PVDF) in *N*-methyl-2-pyrrolidone (NMP). Additional NMP was added to the mixture until a slurry consistency was achieved. The slurries were then manually painted onto 18 mm diameter conductive carbon paper substrates (5.8 mΩ cm^−1^, MSE Supplies) and dried in a vacuum oven for 8 hours at 80 °C. Electrode mass was obtained by subtracting the substrate mass from the total mass after drying and then multiplying by 85%. Approximately 3 mg of working material (VN) was loaded to each electrode.

### Electrochemical setup

Electrochemical characterization was carried out in a three-electrode setup (EL-Cell PAT Series, Aqueous Core) using activated carbon on a stainless steel pseudo-reference electrode and a carbon cloth counter electrode (1000 m^2^ g^−1^, MSE Supplies) (Fig. S1, ESI†). Titanium foil acted as a single-use current collector for both the working and counter electrodes. The working and counter electrodes were separated using two porous separators (21.6 mm × 0.26 mm each) saturated with approximately 400 μL of the electrolyte. The working electrode face and counter electrode were also soaked in the electrolyte overnight prior to assembly.

### Electrochemical measurements

Cyclic voltammetry (CV), galvanostatic charge–discharge (GCD), and potentiostatic electrochemical impedance spectroscopy (EIS) measurements were taken in each electrolyte environment. Each experiment used a fresh electrolyte and fresh working, reference, and counter electrodes. First, open circuit potential (OCP) was recorded until a stable voltage was reached. Next, EIS was conducted at OCP using a frequency range from 200 kHz to 10 mHz at an amplitude of 10 mV. The working voltage window in each electrolyte was then determined using CV by expanding the voltage window at increments of 100 mV at a scan rate of 50 mV s^−1^ until H_2_ and O_2_ onset was reached indicated by a sharp increase in current magnitude; the working window was cut to just before the onset of these reactions (Fig. S2, ESI†). To begin characterizing the material, CV scans were performed at variable scan rates from 2 to 2000 mV s^−1^ (Fig. S3, ESI†) followed by a 1000 cycle stability test at 50 mV s^−1^. Next, to determine the electrochemical surface area (ECSA), CV scans were performed within a 150 mV window of the operating voltage window wherein no redox activity occurred at variable scan rates from 2 to 200 mV s^−1^. Finally, GCD measurements were taken at varying current densities from 0.1 to 50 mA cm^−2^.

### Capacitance calculation

Gravimetric specific capacitance (F g^−1^) values were calculated using:
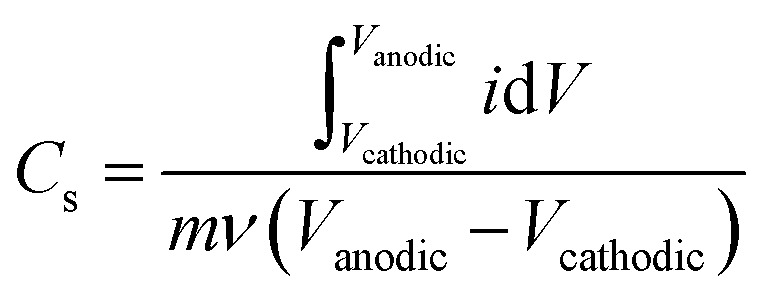
where *V*_cathodic_ (V) and *V*_anodic_ (V) represent cathodic and anodic potential limits, respectively, *i* (A) represents the current, *m* (g) represents the electrode mass, and *ν* (mV s^−1^) represents the scan rate.

From *C*_s_, areal specific capacitance (μF cm^−2^) values were calculated using
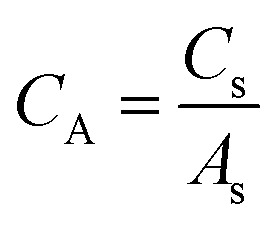
where *A*_s_ (m^2^ g^−1^) represents the physical surface area obtained from N_2_ physisorption analysis.

### Electrochemical surface area (ECSA) calculation

Electrochemical double layer capacitance (*C*_DL_) was measured to determine the difference of ECSA in different electrolytes. Within the operating voltage window for each electrolyte, a 150 mV non-faradaic region was chosen and CVs were run at different scan rates from 2 mV s^−1^ to 200 mV s^−1^ to obtain the corresponding current. Here, the specific double layer capacitance (*C*_SDL_) was estimated to be 50 μF cm^−2^ based on the maximum theoretical specific charge of 50 μC cm^−2^ stored by a double layer capacitor operating under a 1 V operating voltage as reported previously in the literature.^52,53^

The electrochemical surface areas (ECSAs) of VN in different electrolytes were estimated using the relationship given below.
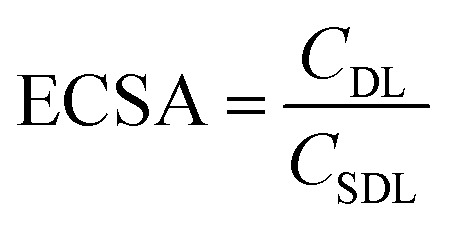


## Results and discussion

**Fig. 1:**
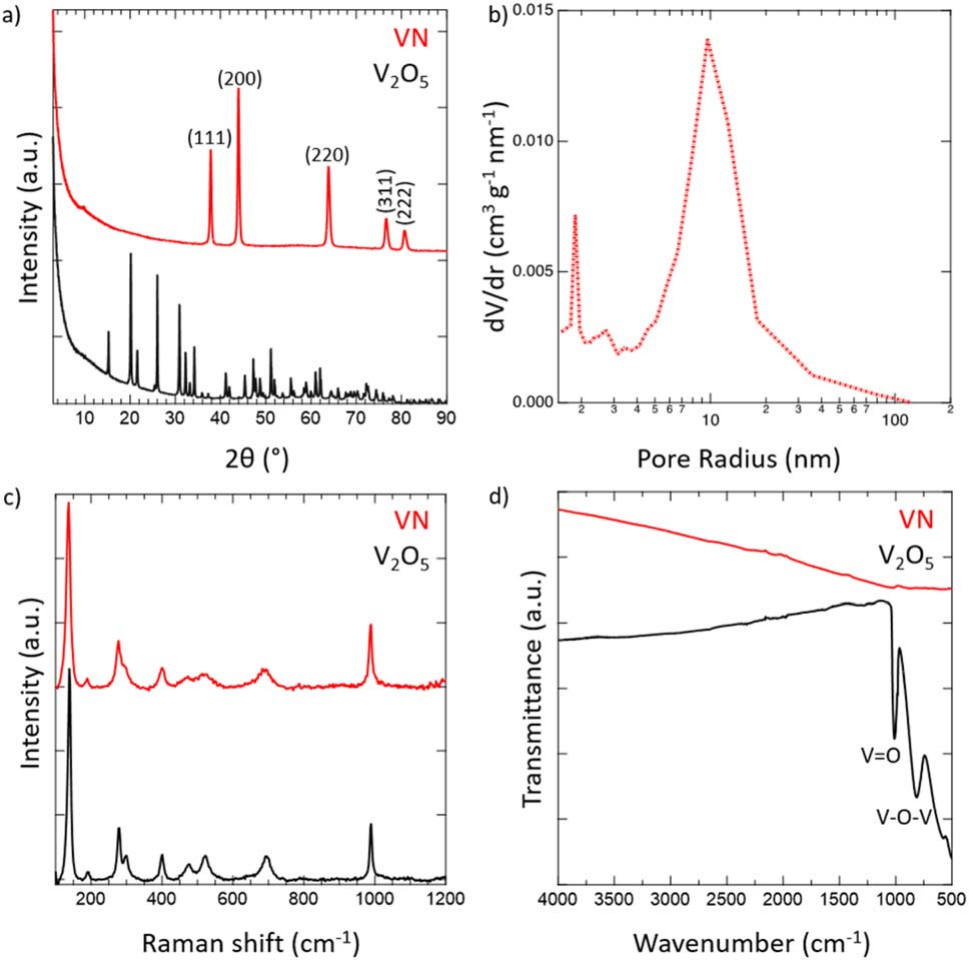
Physical characterization of high surface area passivated VN powder using (a) XRD for bulk characterization, (b) N_2_-physisorption for pore size distribution, and (c) Raman and (d) FTIR spectroscopies for surface characterization.

Crystalline VN powder was synthesized by the reduction of an oxide precursor V_2_O_5_ through a temperature-programmed reaction at 750 °C in an NH_3_ atmosphere. During the reaction, the initial oxide powder changes color from a light orange characteristic of the oxide to black which visually indicates the successful reduction to VN. Following the reaction, the synthesized VN was then cooled to room temperature and passivated with an oxide layer. While it has been shown that passivation of the VN material does affect the electrochemical performance,^54^ the surface was oxidized to better mimic the situations that would be practically utilized for device construction. XRD spectra of both V_2_O_5_ and the passivated VN are provided in Fig. 1a. The lack of oxide peaks in the VN spectrum (red) which are present in that of V_2_O_5_ (black) strongly suggests the successful reduction of the bulk material. Moreover, the emergent peaks in the spectrum of VN belong to crystallographic planes characteristic of the material as reported in previous studies.^55^ The surface morphology was investigated *via* scanning electron microscopy (SEM) (Fig. S4, ESI†). From the SEM images, the porous nature of the material and the surface passivation layer can be clearly seen. Pore size distribution obtained from N_2_ physisorption is shown in Fig. 1b. The average pore size determined by the BJH method was 9.6 nm. The physical surface area calculated by the BET method was 32 m^2^ g^−1^, characteristic of high surface area VN.^56^ The adsorption isotherm and the BET linear regression are reported (Fig. S5, ESI†). Raman spectroscopy was then performed on VN and V_2_O_5_ to characterize the material surface, with both materials producing identical Raman spectra (Fig. 1c). The matching profile of the spectra, combined with the strict surface analysis of Raman spectroscopy, confirms the presence of a surface oxide layer on the material.^57^ These peaks are detected at laser powers as high as 50% indicating that a thick passivation layer was created over the material's surface. Nonetheless, the XRD spectrum of VN proves that the oxide is present on the surface only and does not extend into the bulk of the material. FTIR spectra for both V_2_O_5_ and VN were obtained to characterize the oxide bands present in the passivation layer (Fig. 1d). The transition from the bulk oxide to the surface oxide is easily traced through the disappearance of the V–O–V and V=O peaks (615, 827, and 1020 cm^−1^).^58^ Upon surface reduction *via* amination, the peaks corresponding to the bulk oxide fade drastically in intensity, leaving minimal transmittance in the VN material. By combining the bulk characterization results from XRD spectra, surface characterization results from Raman and FTIR spectra, and the surface area and pore size distribution from N_2_ physisorption, there is strong evidence that high surface area VN was successfully synthesized and passivated.

**Fig. 2:**
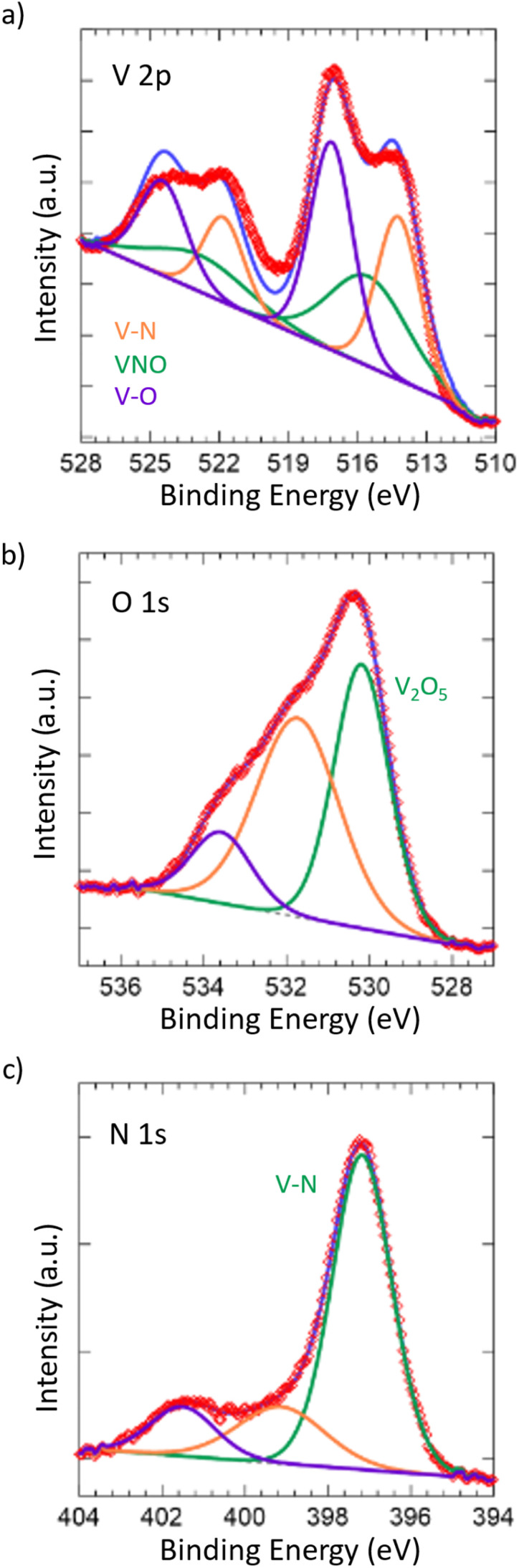
XPS spectra of (a) V 2p, (b) O 1s, and (c) N 1s for the synthesized VN. All spectra were plotted with respect to reference values. The degree of surface oxidation and amount of pristine vanadium nitride structure were tracked according to the V 2p, O 1s, and N 1s spectra.

To further confirm the oxide being limited to the surface of the material, X-ray photoelectron spectroscopy (XPS) was performed (Fig. 2). Taking a look at the V 2p spectrum, the peaks at 517.13 eV and 524.46 eV correspond to the 2p_3/2_ and 2p_1/2_ of the V–O bonding in V_2_O_5_,^59–61^ which is in accordance with our Raman spectrum showing the passivated layer. Additionally, the peaks at 515.53 eV and 522.86 eV correspond to the 2p_3/2_ and 2p_1/2_ of the VNO bonding from VN_*x*_O_*y*_,^55,62^ further indicating the presence of the passivated layer. The O 1s spectrum shows corroboration, where the peak at 530.18 eV corresponds to V_2_O_5_ and the remaining peaks, while unidentified, are likely due to the slight surface oxidation of VN,^63^ which is in accordance with the VN_*x*_O_*y*_ structure that is present. Finally, the peaks at 514.23 eV and 521.83 eV correspond to the 2p_3/2_ and 2p_1/2_ of the VN.^55,63^ This is also in accordance with the peak at 397.17 eV in the N 1s spectrum, which corresponds to the metal–nitride bonding, in this case VN. In contrast to the Raman spectrum, the presence of the VN structure in the surface from XPS is considerable, which can be attributed to the difference in the absorptivity of the material towards visible light and X-rays.

**Fig. 3:**
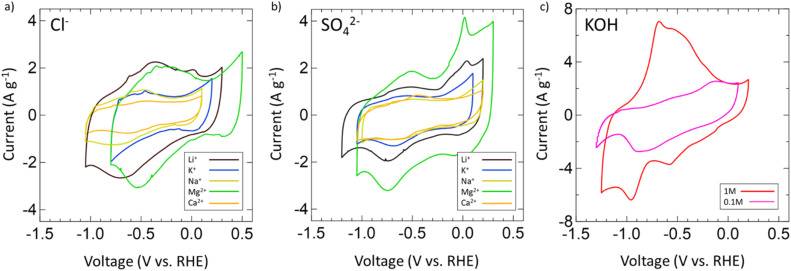
CVs of operating voltage windows for (a) chlorides, (b) sulfates of Mg^2+^ (green), Li^+^ (black), K^+^ (blue), Na^+^ (yellow), and Ca^2+^ (orange) at 50 mV s^−1^. (c) CVs for 1 M (red) and 0.1 M (pink) KOH. All chlorides and sulfates are 1 M concentration except for K_2_SO_4_ (0.5 M), CaSO_4_ (0.06 M), and CaCl_2_ (0.02 M) due to low solubility at room temperature.

After confirming the successful synthesis of VN, its performance as a supercapacitor electrode was assessed first in aqueous 1 M and 0.1 M KOH and then in multiple aqueous salts, specifically chlorides and sulfates of varying cations (Mg^2+^, Ca^2+^, Li^+^, K^+^, Na^+^). For each salt, a 1 M solution was prepared with the exceptions of K_2_SO_4_, CaSO_4_, and CaCl_2_ which have solubilities below 1 M at room temperature. As such, solutions of 0.5 M K_2_SO_4_, 0.02 M CaSO_4_, and 0.06 M CaCl_2_ were prepared. Each electrolyte received a fresh VN electrode as the working electrode and was conditioned by running OCP and EIS centered at OCP (Fig. S6, ESI†). After conditioning, the operating voltage window for VN in each electrolyte was determined by running CVs at a scan rate of 50 mV s^−1^. Fig. 3 provides initial operating voltage windows for aqueous chlorides (Fig. 3a) and sulfates (Fig. 3b) compared against 1 M (red) and 0.1 M (pink) KOH (Fig. 3c) at 50 mV s^−1^. Among both sulfates and chlorides, Mg^2+^ (green) and Li^+^ (black) environments possessed the largest voltage windows. MgCl_2_ and MgSO_4_ achieved respective windows of 1.3 V and 1.35 V while LiCl and Li_2_SO_4_ achieved respective windows of 1.35 V and 1.4 V which are the closest to the characteristic 1.4 V window found in 1 M KOH both experimentally and in previous studies.^64^ Moreover, the voltage windows for Mg^2+^ and Li^+^ environments exist in low cathodic potentials compared to that of KOH which may contribute to their quicker charge and discharge times (Fig. S7, ESI†).

**Fig. 4:**
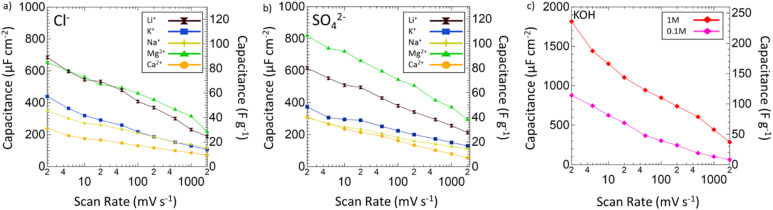
Specific capacitance plotted against scan rate from 2 to 2000 mV s^−1^ of VN in various (a) chlorides and (b) sulfates. Areal specific capacitance (μF cm^−2^) normalized by physical surface area is displayed on the left axis. Gravimetric specific capacitance (F g^−1^) is displayed on the right axis. Capacitances are shown for Li^+^ (black hourglasses), K^+^ (blue squares), Na^+^ (yellow stars), Mg^2+^ (green triangles), and Ca^2+^ (orange circles) systems. (c) Specific capacitance against scan rate for 1 M (red diamonds) and 0.1 M (pink diamonds) KOH.

After the operating voltage was found for each electrolyte, CVs at variable scan rates from 2 to 2000 mV s^−1^ were taken and both gravimetric and areal specific capacitances were calculated for Mg^2+^ (green triangles), Li^+^ (black hourglasses), K^+^ (blue squares), Na^+^ (yellow stars), and Ca^2+^ (orange circles) chlorides (Fig. 4a) and sulfates (Fig. 4b) compared to 1 M (red diamonds) and 0.1 M (pink diamonds) KOH (Fig. 4c). Areal specific capacitances are based on the physical surface area obtained by N_2_ physisorption and were obtained by dividing the gravimetric capacitance by the physical surface area. In each electrolyte, capacitance decreased logarithmically with increasing scan rate indicative of pseudocapacitive charge storage mechanisms for every environment. Despite high capacitances at low scan rates in 1 M and 0.1 M KOH, from 2 to 2000 mV s^−1^ only 16% and 7% of the capacitances were retained, respectively. In 1 M KOH, the specific capacitance dropped from 1816 μF cm^−2^ (236 F g^−1^) to 285 μF cm^−2^ (37 F g^−1^) and in 0.1 M KOH from 877 μF cm^−2^ (114 F g^−1^) to 63 μF cm^−2^ (8 F g^−1^), which is only a slightly better performance than double layer capacitance (50 μF cm^−2^). However, in Mg^2+^ systems, despite lower capacitance at low scan rates compared to KOH, the retained capacitances when moving to higher scan rates are substantially better with 33% in MgCl_2_ and 36% in MgSO_4_. In fact, at 2000 mV s^−1^, MgSO_4_ achieves a specific capacitance of 294 μF cm^−2^ (38 F g^−1^), which is higher than that of 1 M KOH at the same scan rate. This outperformance of 1 M MgSO_4_ compared to 1 M KOH both in terms of capacitance and retention suggests that Mg^2+^ environments are preferable for high-rate charging and discharging.

Next, the electrochemical surface area of VN in each system was evaluated by selecting 150 mV subsections of the operating window CVs where there was no significant redox activity and by running additional CV scans in these regions from 2 to 200 mV s^−1^ (Fig. S8, ESI†). Regions of low redox activity represent low pseudocapacitive contribution better representing double layer capacitive behavior, which can be used to model the electrochemical surface area (Fig. S9, ESI†). When evaluating the electrode surface area for electrochemical applications, BET analysis has limited capabilities. Due to dead pores, the physical surface area of the powder does not directly correspond to the ECSA, which can more accurately predict the electrochemically active and accessible sites, which is an important parameter for selecting supercapacitor electrodes and electrolytes. The ECSAs of VN in the different electrolytes are listed in Table 1.

**Table 1:** Calculated electrochemical surface area of VN in various salt electrolytes and 0.1 M and 1 M KOH. Values were determined from 150 mV subsections of operating window CVs wherein low pseudocapacitive behavior (low redox activity) is present

Electrolyte	Electrochemical surface area (m^2^ g^−1^)
0.5 M K_2_SO_4_	17.7
KCl	11.7
Na_2_SO_4_	12.6
NaCl	18.4
Li_2_SO_4_	16.3
LiCl	22.6
MgSO_4_	11.8
MgCl_2_	25.8
0.02 M CaSO_4_	10
0.06 M CaCl_2_	9.7
1 M KOH	31.1
0.1 M KOH	11.5

**Fig. 5:**
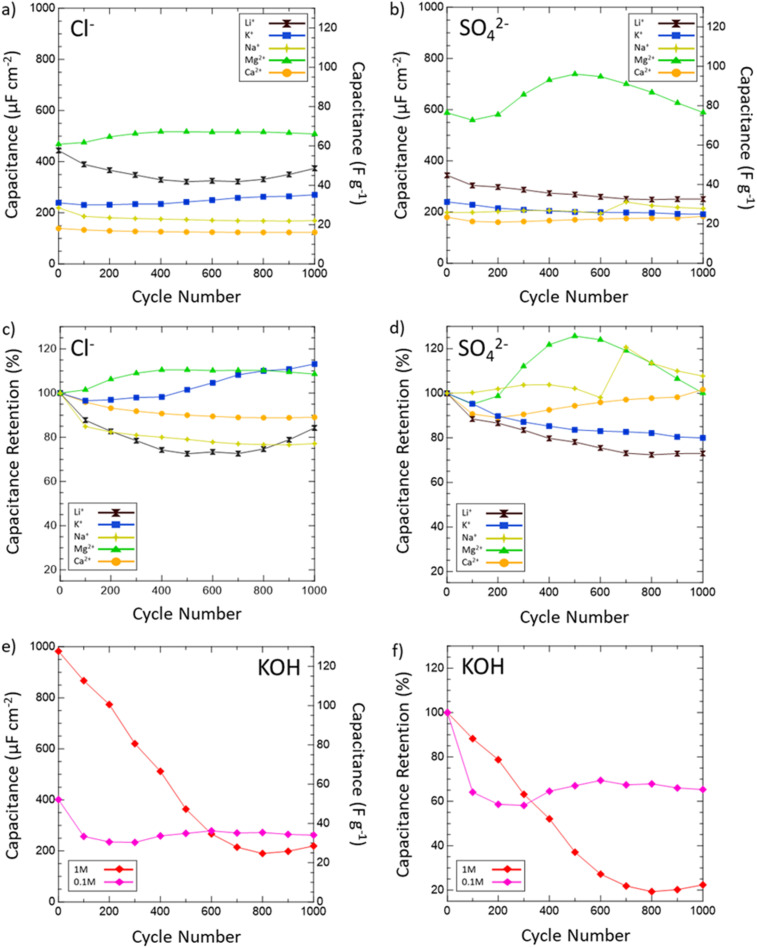
Capacitance values in various salt (a) chlorides and (b) sulfates and retention in (c) chlorides and (d) sulfates of VN after 1000 cycles at 50 mV s^−1^. Areal capacitances are normalized by the physical surface area obtained from N_2_ physisorption. Capacitances are shown for Li^+^ (black hourglasses), K^+^ (blue squares), Na^+^ (yellow stars), Mg^2+^ (green triangles), and Ca^2+^ (orange circles) systems. (e) Capacitance values and (f) retention values in 1 M (red diamonds) and 0.1 M (pink diamonds) KOH. Retention values are determined by dividing the capacitance after every 100 cycles by the 1st cycle capacitance and multiplying by 100.

Then, the electrochemical stability of VN was investigated by cycling 1000 CVs in each electrolyte at 50 mV s^−1^ (Fig. 5). These stability tests assessed both capacitance values over time and percent retention *versus* initial capacitance. After 1000 cycles, capacitance in 1 M KOH drops from 983 μF cm^−2^ (128 F g^−1^) to just 219 μF cm^−2^ (29 F g^−1^) displaying only 22% retention. In 0.1 M KOH, capacitance retention increased to 65% but at the cost of a lower overall capacitance throughout from 401 μF cm^−2^ (52 F g^−1^) to 262 μF cm^−2^ (34 F g^−1^) after 1000 cycles. Meanwhile, capacitance retention in salt environments after 1000 cycles does not drop below 73% (1 M LiCl) and even grows as seen in 1 M KCl (113%), 0.02 M CaSO_4_ (102%), 1 M MgCl_2_ (109%), 1 M Na_2_SO_4_ (peaks at 121% and ends at 108%), and 1 M MgSO_4_ (peaks at 126% and ends at 100%). Moreover, Mg^2+^ environments once again provide the best performance overall in terms of capacitance value and retention with MgCl_2_ surpassing 1 M KOH in capacitance after 400 cycles and MgSO_4_ doing the same after just 300 cycles.

**Fig. 6:**
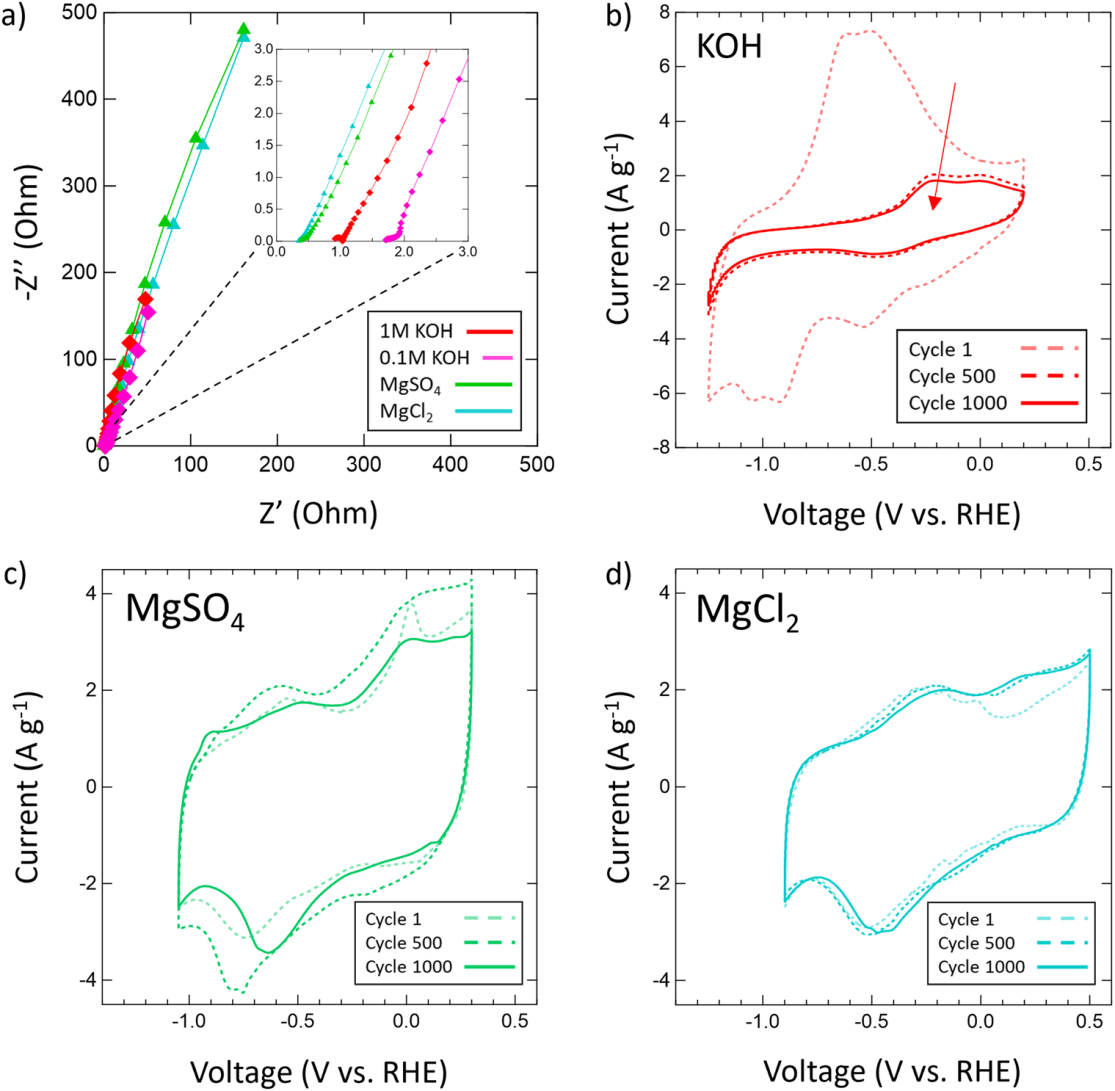
(a) EIS spectra for 1 M KOH (red), 0.1 M KOH (pink), 1 M MgSO_4_ (green) and 1 M MgCl_2_ (cyan) with a frequency range of 200 kHz to 10 mHz and an amplitude of 10 mV; (b) stability CVs of VN in (b) 1 M KOH, (c) MgSO_4_ and (d) MgCl_2_ at 50 mV s^−1^ at cycle 1, cycle 500, and cycle 1000.

From these scan rate and stability capacitance data, the effect of different anions and cations can also be analyzed between different salt solutions. The decision to investigate chlorides and sulfates was influenced by the different oxidation states of the chloride (−1) and sulfate (−2) anions and their different sizes. A wide range of cations were selected to also investigate the influence of cation charge, size, and solubility on the pseudocapacitive mechanism of VN. From the capacitance data obtained, for most cations there seems to be no distinct effect or pattern from changing the anion oxidation state or size on capacitance values or retention. However, among sulfates and chlorides there is a consistent trend in performance influence by the cation used in the order Mg^2+^ > Li^+^ > K^+^ > Na^+^ > Ca^2+^. The high performance of Mg^2+^ systems at higher scan rates and after extensive cycling is substantial enough to outperform KOH systems of equal concentrations over time despite lower initial charge transfer from conditioning EIS data prior to cycling (Fig. 6a). In KOH solutions, the pseudocapacitive mechanism has been extensively studied and reported as shown below:

1 VN_*x*_O_*y*_ + OH^−^ ↔ VN_*x*_O_*y*_‖OH^−^ + VN_*x*_O_*y*_ − OH

which implies a reversible redox mechanism wherein charge is transferred across the electrical double layer *via* hydroxy ion adsorption.^39,41^ However, the extent of this reversibility has previously not been studied through extensive cycling for concentrations of 1 M or above which in this study has been revealed to be poor after many hundreds of cycles and may be attributed to the irreversible oxidation of the oxynitride layer on the passivated VN. This is reflected in the drastic shrinking of CVs for 1 M KOH after 500 and 1000 cycles (Fig. 6b). On the other hand, the remarkable performance of Mg^2+^ systems (Fig. 6c–d) over time implies a different and more reversible pseudocapacitive mechanism as indicated by a consistently larger specific capacitance compared to that of double layer capacitance (50 μF cm^−1^) and high retention after extensive cycling. The proposed mechanism is a 2 e^−^ transfer mechanism as follows:

2 VN_*x*_O_*y*_ + Mg^2+^ + 2e^−^ ↔ Mg[VN_*x*_O_*y*_]

where Mg^2+^ ions interact with the surface oxynitride. This is the most common mechanism reported among aqueous salt systems, and 2 e^−^ transfer mechanisms in Mg^2+^ systems have been previously reported for other early transition metal carbides and nitrides of similar surface structure such as Ti_2_N and Ti_2_C MXenes.^65,66^ A 2 e^−^ charge transfer obviously allows more charge to be transferred for a given charge–discharge cycle which explains the high performance of Mg^2+^ compared to monovalent cations (Li^+^, K^+^, Na^+^). This is likely the same mechanism followed in Ca^2+^ systems as well but high capacitance is limited by the low solubility of calcium salts (CaSO_4_: 0.02 M at 25 °C, CaCl_2_: 0.06 M at 25 °C) which significantly reduces the number of ions available in solution.

## Conclusion

We report the synthesis of interstitial VN powder from a precursor V_2_O_5_ powder through a temperature-programmed reaction. XRD analysis suggests successful reduction of the oxide into VN while N_2_ physisorption analysis verifies a large physical surface area (32 m^2^ g^−1^). Raman spectra reveal the presence of a surface oxide layer indicating successful oxide passivation after synthesis. Electrochemical characterization of the VN was carried out in 1 M and 0.1 M KOH solutions as well as a variety of aqueous salt chlorides and sulfates of Mg^2+^, Ca^2+^, K^+^, Na^+^, and Li^+^. Although VN reports higher capacitance at low scan rates in KOH, a steep drop in capacitance occurs when moving to higher scan rates of 2000 mV s^−1^ leading to low retentions of 16% in 0.1 M KOH (114 to 8 F g^−1^) and only 7% in 1 M KOH (236 to 37 F g^−1^) from 2 to 2000 mV s^−1^. Yet, in aqueous salt environments, specifically Mg^2+^ systems, there is improved capacitance retention of 33% in MgCl_2_ (85 to 28 F g^−1^) and 36% in MgSO_4_ (106 to 38 F g^−1^) when moving to higher scan rates. Capacitance in 1 M KOH was also found to decline sharply after 1000 cycles from 128 to 28 F g^−1^ giving a 22% retention. On the other hand, salt environments provided improved capacitance retention with percent retentions >100% in 1 M KCl (113%), 0.02 M CaSO_4_ (102%), 1 M MgCl_2_ (109%), 1 M Na_2_SO_4_ (peaks at 121% and ends at 108%), and 1 M MgSO_4_ (peaks at 126% and ends at 100%). Mg^2+^ environments also provide the best performance overall in terms of capacitance value and retention with MgCl_2_ surpassing 1 M KOH in capacitance after 400 cycles and MgSO_4_ doing the same after just 300 cycles. Analyzing the capacitance trends across different salt electrolytes led to the conclusion that changes in the anion selection (SO_4_^2−^ and Cl^−^) produced no noticeable effect on performance while changes in cation produced varying performance in the order Mg^2+^ > Li^+^ > K^+^ > Na^+^ > Ca^2+^. The remarkable performance of VN in Mg^2+^ systems is attributed to a 2 e^−^ transfer pseudocapacitive mechanism between Mg^2+^ and the VN oxynitride surface which is a faster and more reversible mechanism than the redox system found in KOH. Current work is underway to unveil the surface behavior and pseudocapacitive mechanism of VN in Mg^2+^ and KOH systems *via in situ*/operando spectroelectrochemical methods. A comprehensive understanding of these two systems can potentially be used to optimize the electrochemical environment of VN to incorporate both the high initial capacitance of KOH systems and the more stable and less corrosive behavior of Mg^2+^ systems.

## Author contributions

James Kasten: conceptualization, methodology, investigation Cheng-Che Hsiao: conceptualization, methodology, resources; Denis Johnson: verification, formal analysis, investigation; Abdoulaye Djire: formal analysis, visualization, project administration.

## Conflicts of interest

There are no conflicts to declare.

## Supplementary Material

NA-005-D2NA00810F-s001
