# Supplementary material for: Superior cyclability of high surface area vanadium nitride in salt electrolytes
Source: Nanoscale Adv. 2023 Jun 9;5(13):3485–93. doi: 10.1039/d2na00810f (PMC10295220; doi:10.1039/d2na00810f)
Supplement: NA-005-D2NA00810F-s001 [file NA-005-D2NA00810F-s001.pdf]

Electronic Supplementary Material (ESI) for Nanoscale Advances.
This journal is © The Royal Society of Chemistry 2023

Supporting Information

Superior Cyclability of High Surface Area Vanadium Nitride in Salt Electrolytes

James Kasten[1], Cheng-Che Hsiao[1], Denis Johnson[1], and Abdoulaye Djire[1,2*]

[1]Artie McFerrin Department of Chemical Engineering, Texas A&M University, College Station, TX 77843, USA

[2]Department of Materials Science & Engineering, Texas A&M University, College Station, TX 77843, USA

*Corresponding Author: adjire@tamu.edu

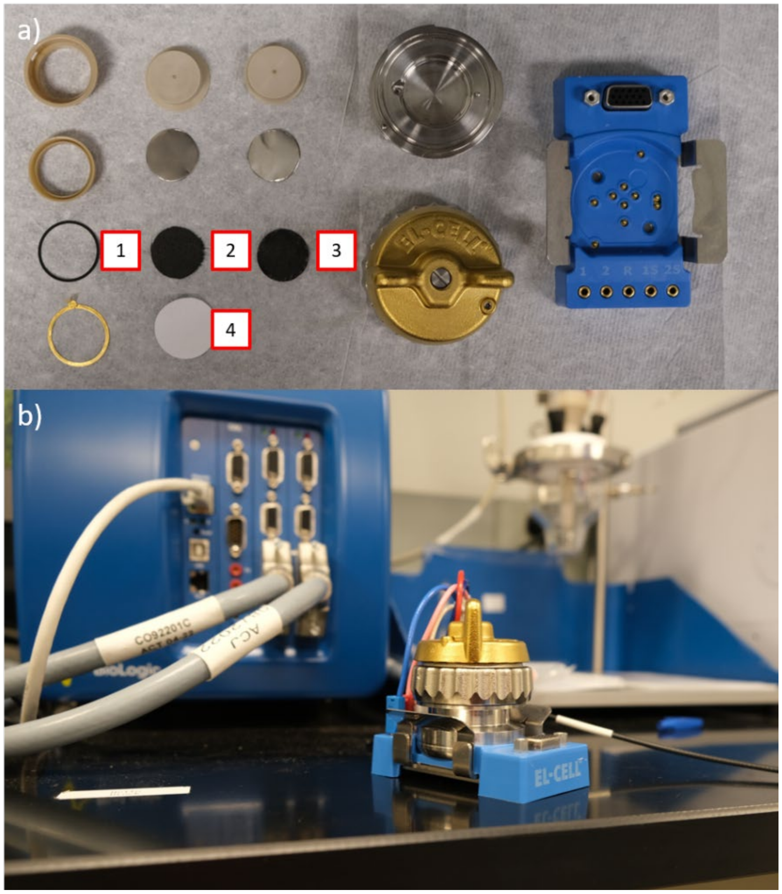

Figure S1. a) Disassembled 3-electrode electrochemical cell (EL-Cell, PAT-Cell) including the reference electrode (1), counter electrode (2), working electrode (3), and separator (4). b) Electrochemical cell connected to the potentiostat for current study.

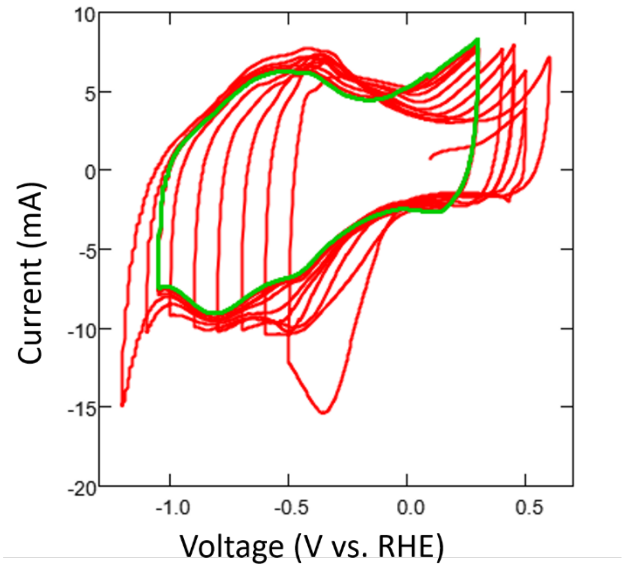

Figure S2. Example cyclic voltammogram (CV) scans after EIS conditions of VN in $MgSO_4$ used to find the operating voltage window. The green CV is the final scan which represents the set working voltage.

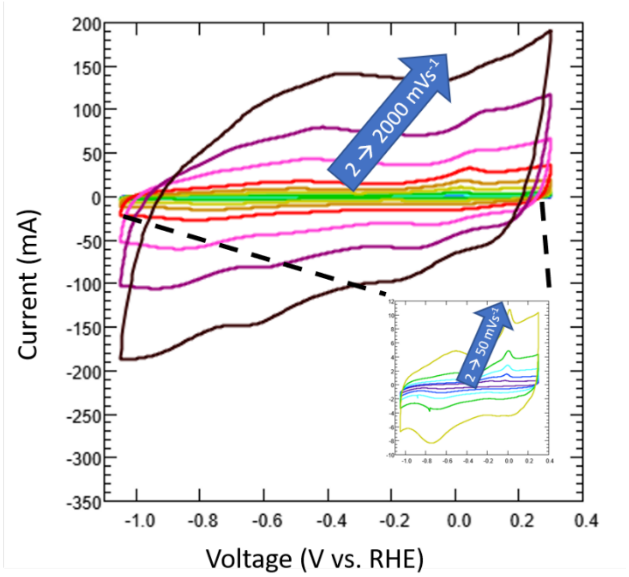

Figure S3. CV scans of VN in $MgSO_4$ at different scan rates from 2 to 2000 mV s^{-1}. The inset is a zoomed in graphs showing scan rates 2 to 50 mV s^{-1}. Scan rates used were 2, 5, 10, 20, 50, 100, 200, 500, 1000, and 2000 mV s^{-1}.

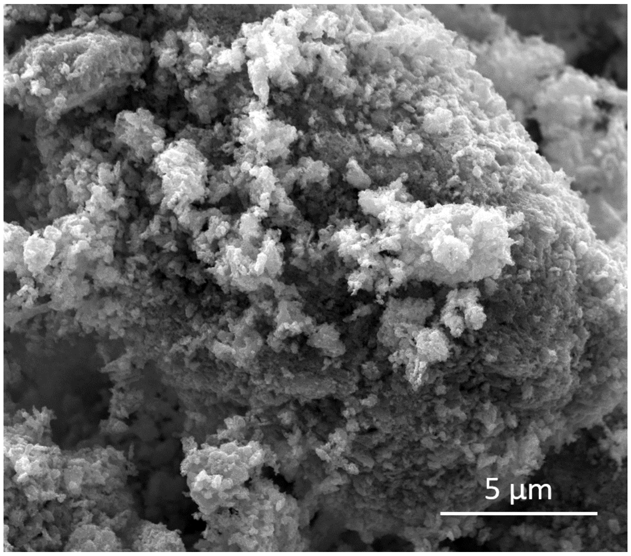

Figure S4. SEM image of the as-prepared VN material following temperature programmed reduction of V_2O_5 in NH_3 atmosphere followed by passivation via 1% O_2/N_2.

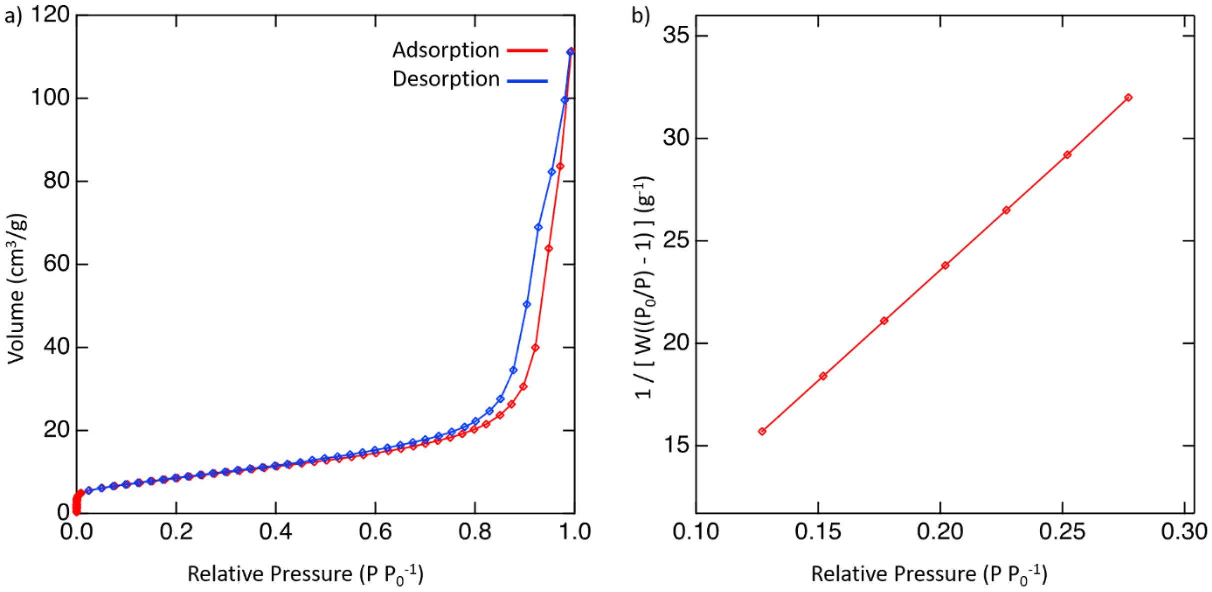

Figure S5. N_2-physisorption analysis of vanadium nitride. a) Adsorption and desorption isotherms. b) Data of BET linear fitting method.

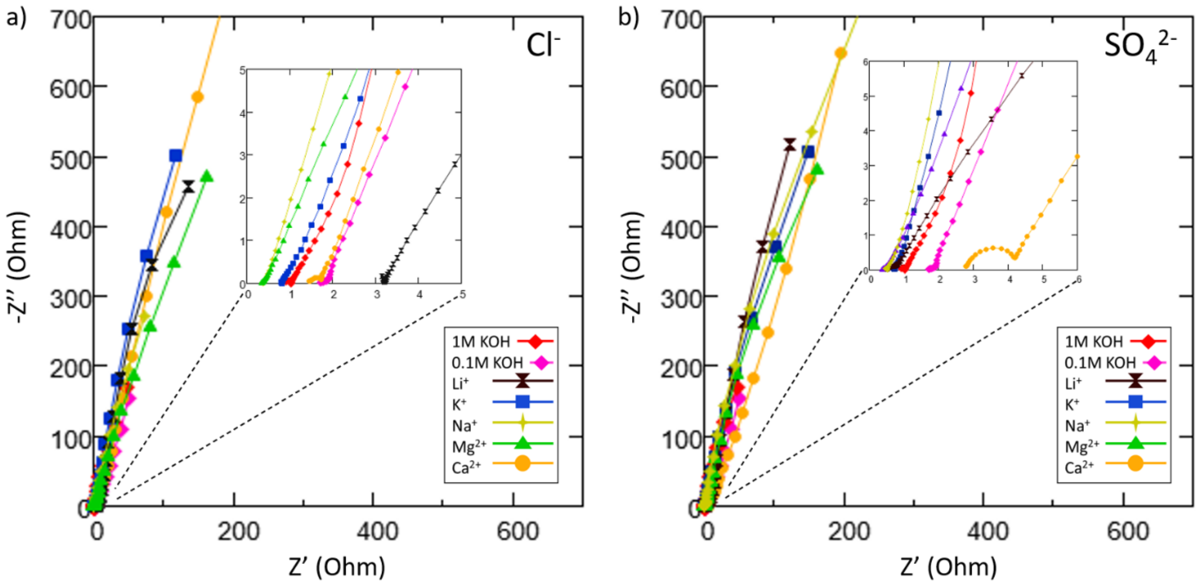

Figure S6. Conditioning electrochemical impedance spectroscopy (EIS) data for VN in various a) chlorides and b) sulfates as well as 1M and 0.1M KOH. EIS was performed immediately after OCP measurement in each electrolyte at OCP using a frequency range of 200 kHz to 10 mHz and a small amplitude of 10 mV.

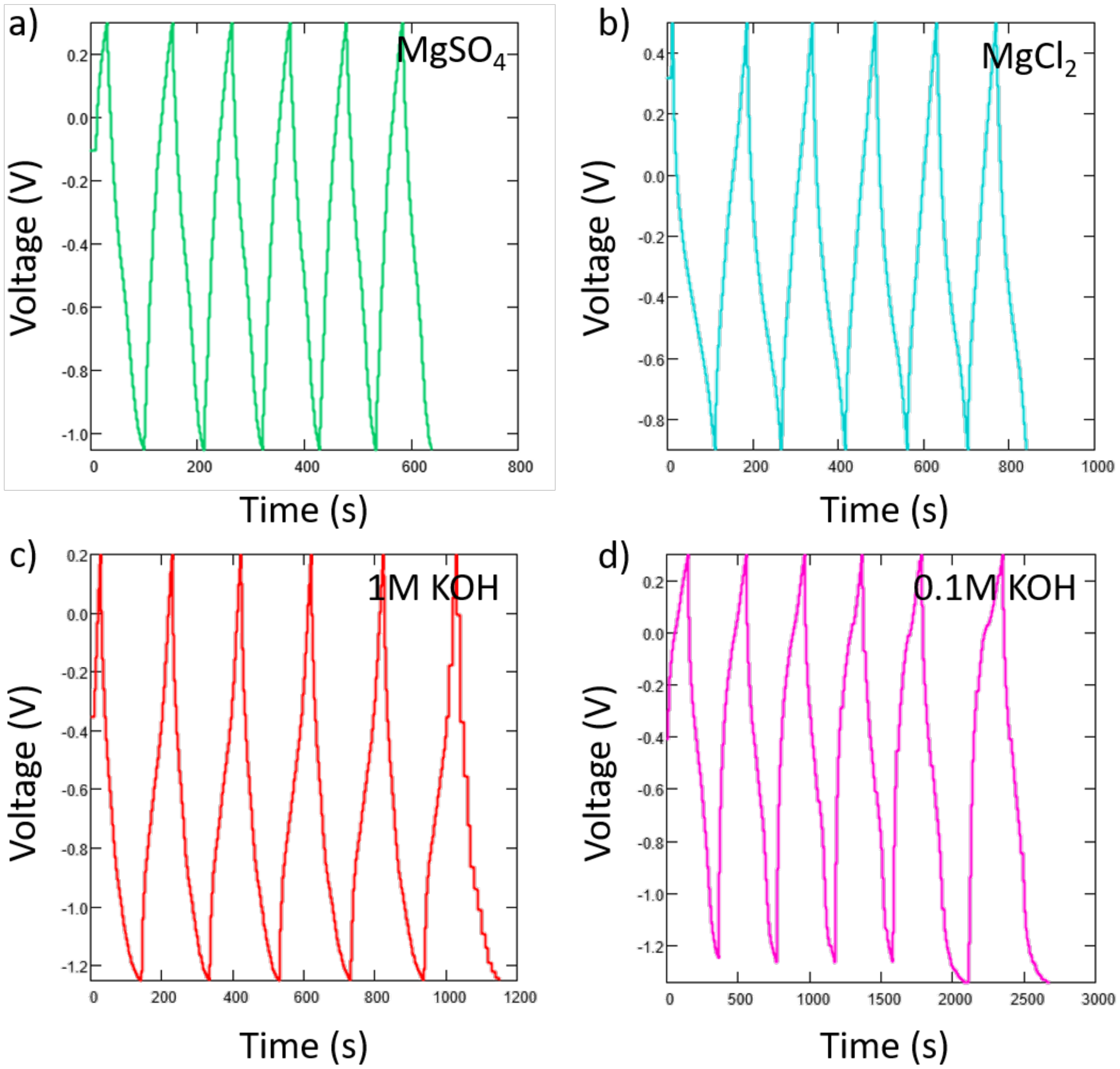

Figure S7. Galvanostatic Charge Discharge (GCD) curves for VN in a) 1M $MgSO_4$ b) 1M $MgCl_2$ c) 1M KOH and d) 0.1M KOH each at an applied current of 1 mA cm^{-1}.

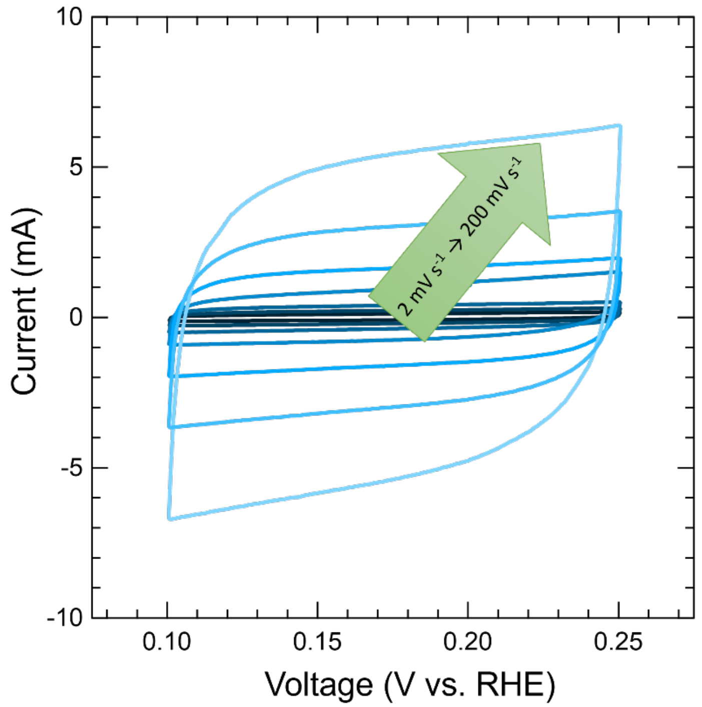

Figure S8. Example of CV curves (from 1M $MgCl_2$ system) used for ECSA calculations.

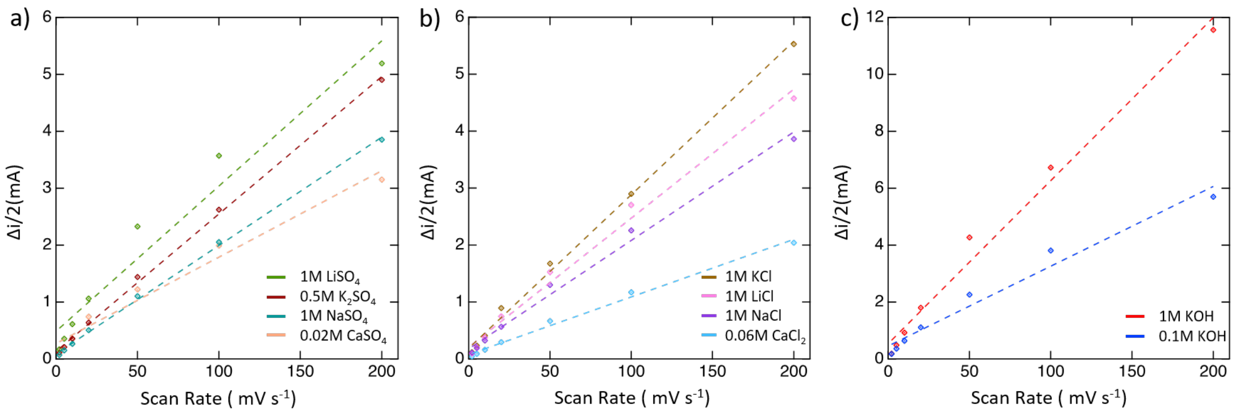

Figure S9. Linear fitting for the calculation of ECSA in different a) sulfate, b) chloride, and c) KOH electrolytes.
